# Development and validation of nomograms for predicting the risk of central lymph node metastasis of solitary papillary thyroid carcinoma of the isthmus

**DOI:** 10.1007/s00432-023-05146-7

**Published:** 2023-08-20

**Authors:** Yonghao Li, Xuefei Gao, Tiantian Guo, Jing Liu

**Affiliations:** 1 The First Clinical Medical College of Shanxi Medical University, Taiyuan, 030001 Shanxi China; 2 Department of Thyroid Surgery, The First Hospital of Shanxi Medical University, 85 South Jiefang Road, Taiyuan, 030001 Shanxi China

**Keywords:** Papillary thyroid carcinoma of the isthmus (PTCI), Solitary, Lymph node metastasis (LNM), Nomograms, Risk stratification

## Abstract

**Background:**

This study was conducted to develop nomograms and validate them by assessing risk factors for the development of central lymph node metastasis (CLNM) in patients with solitary papillary thyroid carcinoma of the isthmus (PTCI) for predicting the probability of CLNM.

**Methods:**

Demographic and clinicopathological variables of patients with solitary papillary thyroid carcinoma (PTC) from May 2018 to May 2023 at the First Hospital of Shanxi Medical University were retrospectively analyzed, and the lobar group and the isthmus group were divided according to tumor location. Patients with the same sex, age difference of less than 3 years, and equal gross tumor diameter were selected from the lobar group and compared with the paraisthmic tumor group. Independent risk factors were determined using univariate and multivariate logistic regression analysis. On this basis, clinical predictive nomograms were developed and validated.

**Results:**

Clinical data from 326 patients with solitary PTCI and 660 cases of solitary lobar PTC were used for analysis in our study. The incidence of solitary tumors CLNM located in the median isthmus, paracentral isthmus, and lobes was 69.8%, 40.9%, and 33.6%, respectively. Statistical analysis revealed that gender, age, isthmus location, maximum nodal diameter, the presence of possible CLNM in advance on preoperative ultrasound, chronic lymphocytic thyroiditis, and the lymphocyte/monocyte ratio were independent risk factors for preoperative CLNM in patients with solitary PTCI. Age, isthmus location, chronic lymphocytic thyroiditis, gross tumor diameter, presence of intraoperative extrathyroidal extension, and presence of metastasis in the Delphian lymph node on frozen section were independent risk factors for intraoperative CLNM. The concordance indices of nomograms for preoperative and intraoperative are 0.871 and 0.894 in the training set and 0.796 and 0.851 in the validation set, calibration curve and decision curve analysis also demonstrated the strong reliability and clinical applicability of this clinical prediction model.

**Conclusion:**

In this study, we concluded that solitary PTCI is more aggressive compared to solitary lobar PTC, and we constructed nomograms and risk stratification to accurately identify patients with solitary PTCI who are at high risk of developing CLNM, which will help clinicians in personalized decision making.

## Introduction

The incidence of papillary thyroid carcinoma (PTC) has increased rapidly worldwide in recent years (Siegel et al. 2023), and although its prognosis is good, the risk of postoperative recurrence is still high because PTC is prone to lymph node metastasis (LNM). However, due to the specific location of the tumor, papillary thyroid carcinoma of the isthmus (PTCI) is more likely to have pathological features such as multifocal tumor, capsular breach, extrathyroidal extension (ETE) and LNM (Lyu et al. 2021; Lee et al. 2010; Luo et al. 2020). Although PTCI is more aggressive than lobar PTC, due to the low incidence and lack of large-scale clinical studies, the current consensus and guidelines do not specify the characteristics of LNM and the extent of surgery for PTCI (Haugen et al. 2016). To reduce the risk of postoperative recurrence in patients with PTCI, total thyroidectomy (TT) + bilateral central lymph node dissection (BCLND) is the more common procedure chosen by clinicians, but the postoperative medication burden and postoperative complication rate of patients under this procedure are high, so some scholars suggested that the scope of surgical resection can be reduced for PTCI with smaller diameter to reduce the negative impact of surgery on patients and concluded that the prognosis is not significantly different from that of the conventional procedure (Huang et al. 2022a, b; Seo et al. 2021). Therefore, it is necessary to explore the central lymph node metastasis (CLNM) pattern and construct a prediction model to find a more suitable surgical range for PTCI patients.

In this study, we retrospectively analyzed the preoperative relevant tests and clinicopathological features of solitary PTCI and solitary lobar PTC with the aim of assessing the risk factors for CLNM in patients with solitary PTCI and thus exploring the scope of surgery for PTCI. In addition, we constructed and validated nomograms and risk stratification to quantitatively assess the risk of CLNM in patients preoperatively and intraoperatively, so that surgeons can refer to them in clinical practice.

## Materials and methods

### Study population

This study retrospectively analyzed patients with solitary PTC who underwent surgery for the first time from May 2018 to May 2023 at the First Hospital of Shanxi Medical University. All patients underwent high-resolution ultrasound and neck CT preoperatively and were diagnosed with solitary PTC by postoperative pathological examination.

**Fig. 1:**
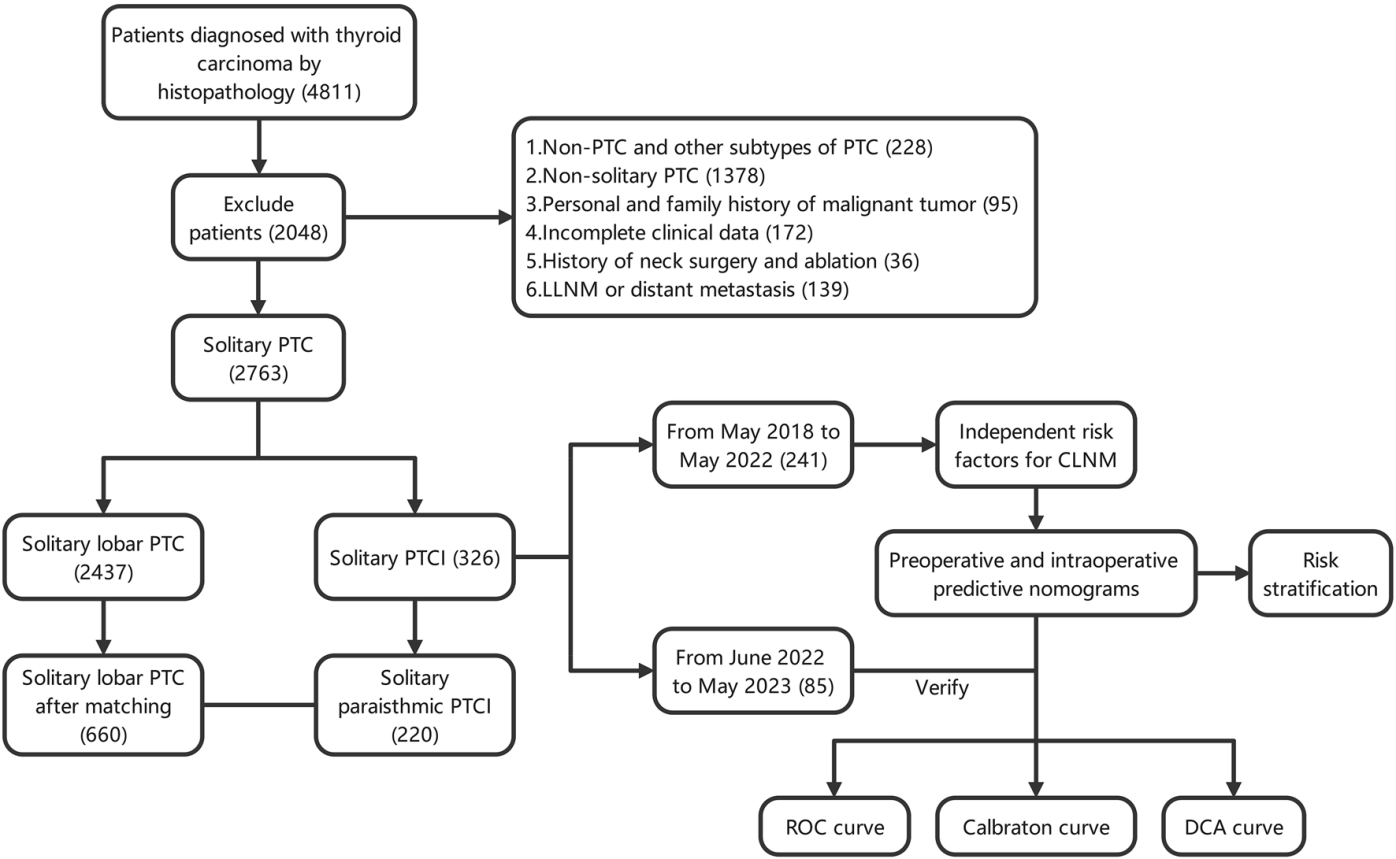
The flow chart of patients enrolled in this study

Figure 1 shows the flow chart of patients enrolled in this study. Based on the location of the tumor, we divided the patients into the PTCI group and the lobar PTC group. Patients included in this study met the following criteria: (1) solitary PTC; (2) no personal history of other malignancies and history of neck surgery; and (3) complete clinical information. The following patients were excluded: (1) patients with non-PTC and non-classical subtype of PTC; (2) patients with multifocal PTC; (3) history of previous neck surgery, radiation; (4) presence of lateral lymph node metastasis (LLNM) or distant metastasis; (5) family history of thyroid carcinoma or presence of previous history of malignancy; (6) incomplete clinical data or missing follow-up. Because of the large difference in the number of patients between the paraisthmic group and the lobar PTC group, to compare the two more efficiently, we matched them according to the following criteria to exclude the interference of some confounding factors, namely: (1) same sex; (2) age difference within 3 years; and (3) same gross tumor diameter. Therefore, 986 patients were finally included in this study, including 326 patients with PTCI, and according to the order of patient admission, we included 241 patients admitted for treatment from May 2018 to May 2022 as the training group and 85 patients admitted for treatment from June 2022 to May 2023 as the validation group, with significant differences in all relevant variables between the two groups shown in Table 1.

**Table 1:** Demographic and clinical characteristics of Training cohort and Validation cohort

**Variables**	Overall(N=326)		*P* value
	Training cohort(N=241)	Validation cohort(N=85)	
**Age (years)**			0.232
≤40	78(32.4%)	34(40.0%)	
＞40	163(67.6%)	51(60.0%)	
**Sex**			0.892
Female	194(80.5%)	69(81.2%)	
Male	47(19.5%)	16(18.8%)	
**BMI**			0.413
Normal	149(61.8%)	58(68.2%)	
Overweght	75(31.1%)	20(23.5%)	
obesity	17(7.1%)	7(8.2%)	
**CLT**			0.489
Presence	68(28.2%)	28(32.9%)	
Absence	173(71.8%)	57(67.1%)	
**Location**			0.477
Paraisthmic isthmic tumor	160(66.4%)	60(70.6%)	
Median isthmic tumor	81(33.6%)	25(29.4%)	
**Maximum tumor size**			0.856
≤0.4	15(6.2%)	5(5.9%)	
(0.4-0.7]	96(39.8%)	33(38.8%)	
(0.7-1**]**	73(30.3%)	23(27.1%)	
＞1	57(23.7%)	24(28.2%)	
**Tumor volume**			0.537
Mean (SD)	0.46(1.76）	0.33(0.57）	
**Gross maximum tumor size**			0.395
Mean (SD)	0.68(0.36）	0.72(0.41）	
**PLR**			0.083
Mean (SD)	140.00(44.49）	149.82(45.22)	
**NLR**			0.397
Mean (SD)	1.83(0.79)	1.75(0.77)	
**LMR**			0.944
Mean (SD)	5.37(1.61)	5.36(1.57)	
**SII**			0.157
Mean (SD)	447.60(218.62)	490.59(294.1)	
**Dyslipidemia**			0.705
Absence	108(44.8%)	36(42.4%)	
Presence	133(55.2%)	49(57.6%)	
**Cr**			0.348
Mean (SD)	60.72(11.29)	59.41(10.27)	
**Tg**			0.248
Mean (SD)	29.22(56.05)	21.77(32.87)	
**TSH**			0.328
Mean (SD)	4.68(16.87)	2.89(1.57)	
**ETE detected during US**			0.129
Presence	13(5.4%）	9(10.6%）	
Absence	228(94.6%）	76(89.4%）	
**Probable CLNM detected during US**			0.576
Presence	30(12.4%）	13(15.3%）	
Absence	211(87.6%）	72(84.7%）	
**ETE detected during surgery**			0.984
Presence	74(30.7%)	26(30.6%)	
Absence	167(69.3%)	59(69.4%)	
**CLNM**			0.952
Presence	121(50.2%)	43(50.6%)	
Absence	120(49.8%)	42(49.4%)	
**DLNM**			0.369
Presence	32(13.3%)	15(17.6%)	
Absence	209(86.7%)	70(82.4%)	

Data notes: *BMI* body mass index, *CLT* chronic lymphocytic thyroiditis, *PLR* platelet/lymphocyte ratio, *NLR* neutrophil/lymphocyte ratio, *LMR* lymphocyte/monocyte ratio,SII the systemic immune-inflammation index, *Tg* Thyroglobulins, *TSH* Thyroid stimulating hormone, *ETE* extrathyroidal extension, *CLNM* central lymph node metastasis, *US* Ultrasound, *DLNM *Delphian lymph node metastasis

### Preoperative examination

In this study, two ultrasonographers with more than 10 years of clinical experience performed ultrasound examinations of suspicious thyroid nodules and cervical lymph nodes to obtain the size of the suspicious nodules and determine the presence of possible ETE and CLNM. The maximum transverse diameter (a), anteroposterior diameter (b), and longitudinal diameter (c) of each tumor were measured by ultrasound, and the volume of each tumor was calculated according to the spherical volume calculation formula, i.e. : V = π/6 × [(a+b+c)/3]^3^. We routinely perform preoperative ultrasound-guided fine-needle aspiration biopsy to further confirm the diagnosis. In this study, two vertical virtual lines were drawn from the lateral border of the trachea to the skin surface, and a PTCI was defined if the intersection of the longest and shortest diameter of the tumor was located between the two lines, and a median PTCI was defined if the mass was located within the middle 1/3 of the isthmus, and a paracentral PTCI was defined if the opposite was true. Body mass index (BMI) was calculated and divided into normal (BMI < 25 kg/m^2^), overweight (25kg/m^2^ ≤ BMI < 30 kg/m^2^), and obese (BMI ≥ 30 kg/m^2^) groups. Meanwhile, we routinely obtained information on patients' blood fat, thyroid function, blood cell analysis, and creatinine by performing preoperative laboratory tests on their serum. Patients are considered dyslipidemic when their low density lipoprotein cholesterol or triglycerides are outside the upper limit of the normal range with or without a decrease in high density lipoprotein cholesterol. Chronic lymphocytic thyroiditis (CLT) is defined by elevated levels of thyroid peroxidase antibodies and thyroglobulin antibodies or diffuse changes in the gland suggested by ultrasound. Using the results of the patient's preoperative blood cell analysis, we calculated the platelet/lymphocyte ratio (PLR), neutrophil/lymphocyte ratio (NLR), lymphocyte/monocyte ratio (LMR), the systemic immune-inflammation index (SII).

### Surgical procedures, histopathologic examination

For all patients with lobar PTC, lobectomy with unilateral central lymph node dissection (UCLND) is performed, while TT + BCLND is performed for patients with PTCI. The UCLND includes the Delphian lymph node (DLN), the pretracheal lymph node, and the affected paratracheal lymph node, while the BCLND removes the DLN, pretracheal and bilateral paratracheal lymph nodes. The surgeries were performed by surgeons with at least 20 years of experience in thyroid surgery. Intraoperative ETE was detected as intraoperative tumor invasion through the thyroid capsule into the soft tissues surrounding the thyroid gland and involvement of peripheral structures such as the strap muscles. High-volume CLNM was defined as ≥5 positive lymph nodes on pathological examination. All pathology specimens were microscopically examined and cross-checked by two or more experienced pathologists.

### Postoperative hypoparathyroidism

Serum parathyroid hormone and blood calcium concentrations were routinely performed on the first postoperative day in patients with PTCI after TT + BCLND. Transient hypoparathyroidism was defined as serum parathyroid hormone < 15 pg/ml or blood calcium concentration < 2.20 mmol/L during hospitalization and return to normal within 6 months. Patients who still required calcium supplementation 6 months after surgery were diagnosed with permanent hypoparathyroidism.

### Statistical analysis

All statistical analyses for this study were performed using R software (Version 4.2.2, R Foundation for Statistic Computing, Austria) (http://www.r-project.org/) and SPSS (Version 25, IBM, USA). Continuous variables were expressed as mean ± standard deviation (SD) or median and interquartile, and categorical variables were reported as number and percentage, comparing clinical information related to median PTCI, paracentral PTCI, and lobar PTC. In the univariate analysis, χ^2^ test, independent* t* test, and nonparametric rank sum test were used to compare relevant variables and to derive risk factors for the development of CLNM in patients with PTCI preoperatively and intraoperatively. Variables with *P* values < 0.1 in the univariate analysis were screened for *P* values < 0.05 using multivariate logistic regression analysis (stepwise forward method). On this basis, the RMS package in R software was used to construct predictive nomograms of preoperative and intraoperative CLNM, and the consistency index was used to evaluate the predictive performance of the model. The receiver operating characteristic (ROC) curve was plotted to show the predictive ability of the model, and the area under curve (AUC) was listed, with higher AUC representing better predictive ability. In addition, calibration curve and decision curve analysis (DCA) were used to assess the clinical value of the nomograms. *P* value < 0.05 (bilateral) was statistically significant. Based on the risk scores of each factor in the preoperative and intraoperative CLNM prediction nomograms, we divided into four risk subgroups by determining the cutoff values: low-risk group, medium-risk group, high-risk group, and extremely high-risk group.

## Results

### Comparison of clinicopathological characteristics of solitary median PTCI, solitary paracentral PTCI, and solitary lobar PTC

As shown in Table 2, We compared 220 patients with solitary paracentral PTCI with 660 patients with solitary lobar PTC of the same sex, age difference within 3 years and gross tumor diameter, and concluded that the incidence of CLNM in solitary median PTCI, solitary paracentral PTCI, and solitary lobar PTC was 69.8%, 40.9%, and 33.6%, and the incidence of ETE was 42.5%, 25.0%, and 14.1%, and the incidence of high-volume CLNM was 26.4%, 11.4%, and 6.8%.

**Table 2:** Demographic and clinical characteristics of PTCI and lobar PTC

Variables	Median isthmic tumor (N = 106)	Left isthmic tumor (N = 107)	Right isthmic tumor (N = 113)	*P* value	Paraisthmic tumor (N = 220)	Lobar tumor (N = 660)	*P* value
Age (years)				0.54			0.192
≤ 40	32 (30.2%)	38 (35.5%)	42 (37.2%)		80 (36.4%)	238 (36.1%)	
> 40	74 (69.8%)	69 (64.5%)	71 (62.8%)		140 (63.6%)	422 (63.9%)	
Sex				0.338			0.918
Female	82 (77.4%)	91 (85.0%)	90 (79.6%)		181 (82.3%)	543 (82.3%)	
Male	24 (22.6%)	16 (15.0%)	23 (20.4%)		39 (17.7%)	117 (17.7%)	
BMI				0.101			0.024
Normal	68 (64.2%)	70 (65.4%)	69 (61.1%)		139 (63.2%)	376 (57.0%)	
Overweight	33 (31.63%)	24 (22.4%)	38 (33.6%)		62 (28.2%)	247 (37.4%)	
Obesity	5 (4.7%)	13 (12.1%)	6 (5.3%)		19 (8.6%)	37 (5.6%)	
Gross maximum tumor size				< 0.001			1
< 1 cm	67 (63.2%)	92 (86.0%)	103 (91.2%)		195 (88.6%)	585 (88.6%)	
≥ 1 cm	39 (36.8%)	15 (14.0%)	10 (8.8%)		25 (11.4%)	75 (11.4%)	
ETE				0.006			< 0.001
Presence	45 (42.5%)	26 (24.3%)	29 (25.7%)		55 (25.0%)	567 (85.9%)	
Absence	61 (57.5%)	81 (75.7%)	84 (74.3%)		165 (75.0%)	93 (14.1%)	
CLNM				< 0.001			0.061
Presence	74 (69.8%)	46 (43.0%)	44 (38.9%)		90 (40.9%)	222 (33.6%)	
Absence	32 (30.2%)	61 (57.0%)	69 (61.1%)		130 (59.1%)	438 (66.4%)	
High-volume CLNM				0.002			0.031
Presence	28 (26.4%)	10 (9.3%)	15 (13.3%)		25 (11.4%)	45 (6.8%)	
Absence	78 (73.6%)	97 (90.7%)	98 (86.7%)		195 (88.6%)	615 (93.2%)	
DLNM				0.32			0.001
Presence	23 (21.7%)	11 (10.3%)	13 (11.5%)		24 (10.9%)	30 (4.5%)	
Absence	83 (78.3%)	96 (89.7%)	100 (88.5%)		196 (89.1%)	630 (95.5%)	
Pretracheal LN metastasis				0.34			0.001
Presence	19 (17.9%)	8 (7.5%)	10 (8.8%)		18 (8.2%)	17 (2.6%)	
Absence	87 (82.1%)	99 (92.5%)	103 (91.2%)		202 (91.8%)	643 (97.4%)	
Left paratracheal LN metastasis				< 0.001			0.033
Presence	48 (47.1%)	37 (34.6%)	13 (11.5%)		50 (22.7%)	108 (16.4%)	
Absence	54 (52.9%)	70 (65.4%)	100 (88.5%)		170 (77.3%)	552 (83.6%)	
LN-arRLN metastasis				< 0.001			0.659
Presence	47 (44.3%)	12 (11.2%)	29 (25.7%)		41 (18.6%)	132 (20.0%)	
Absence	59 (55.7%)	95 (88.8%)	84 (74.3%)		179 (81.4%)	528 (80.0%)	
LN-prRLN metastasis				0.015			0.921
Presence	13 (12.3%)	3 (2.8%)	6 (5.3%)		9 (4.1%)	26 (3.9%)	
Absence	93 (87.7%)	104 (97.2%)	107 (94.7%)		211 (95.9%)	634 (96.1%)	
Parathyroid autotransplantation				0.503			0.021
Presence	12 (11.3%)	16 (15.0%)	19 (16.8%)		35 (15.9%)	67 (10.2%)	
Absence	94 (88.7%)	91 (85.0%)	94 (83.2%)		185 (84.1%)	593 (89.8%)	
Transient hypoparathyroidism	29 (27.4%)	36 (33.6%)	34 (30.1%)	–			
Permanent hypoparathyroidism	2 (1.9%)	3 (2.8%)	3 (2.7%)	–			

*PTCI* papillary thyroid carcinoma of the isthmus, *PTC* papillary thyroid carcinoma, *BMI* body mass index, *ETE* extrathyroidal extension, *CLNM* central lymph node metastasis, *DLNM* Delphian lymph node metastasis, *LN* lymph node, *LN-arRLN* the lymph node anterior to the right recurrent laryngeal nerve, *LN-prRLN* the lymph node posterior to the right recurrent laryngeal nerve

### Clinical and ultrasonographic characteristics of patients with solitary PTCI

Among all patients in this study, 263 (80.6%) were female patients, with a mean age at diagnosis of 45.6 years ± 10.83 (20–73 years), a mean BMI of 24.38 ± 3.65 (17.01–35.63), and a mean preoperative ultrasound suggested maximum tumor diameter of 0.89 cm ± 0.39 (0.18–4.51 cm); 182 patients (55.8%) had dyslipidemia, 96 patients (29.4%) had CLT, 23 patients (7.0%) had preoperative ultrasound suggestive of ETE, 100 patients (30.6%) had ETE detected intraoperatively, and 164 patients (50.3%) had CLNM, of which 52 patients (32.3%) had high-volume CLNM.

### Development of preoperative and intraoperative nomograms for predicting CLNM in patients with solitary PTCI

**Fig. 2:**
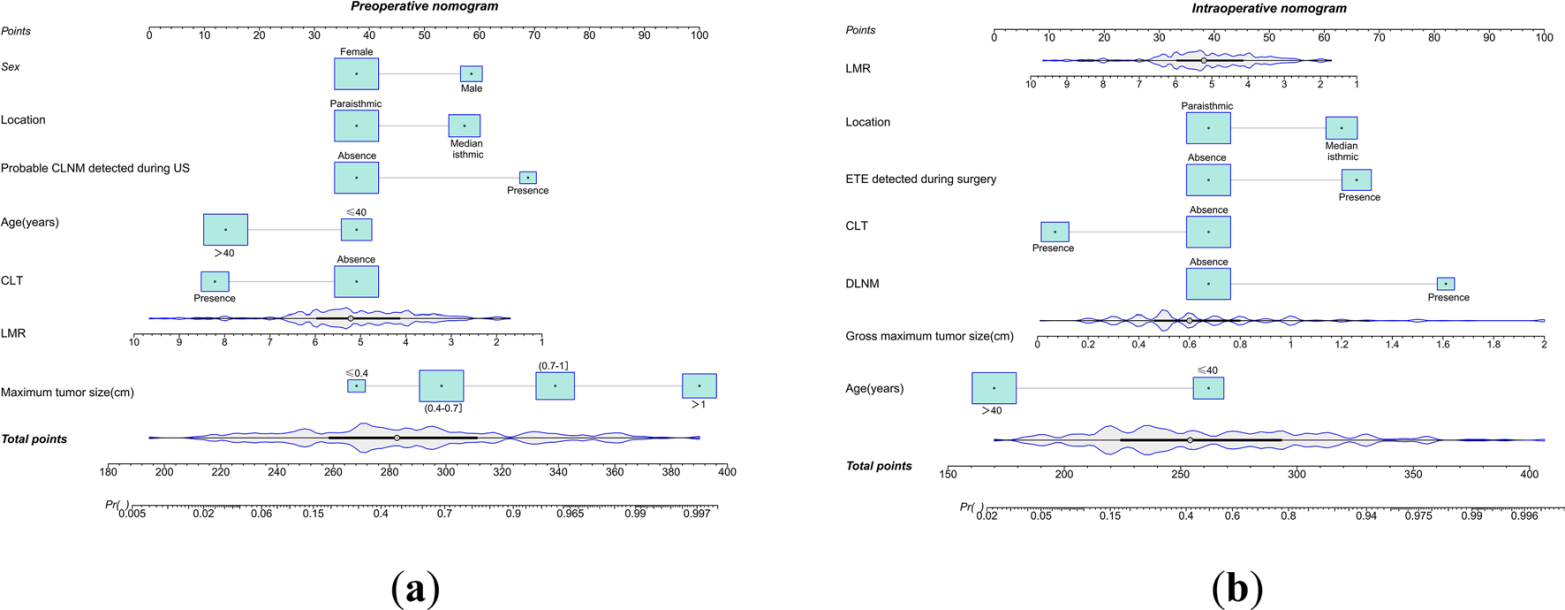
**a** Preoperative CLNM prediction nomograms; **b** intraoperative CLNM prediction nomograms

As shown in Tables 3, 4, the univariate and multivariate analyses concluded that gender, age, isthmus tumor location, maximum nodal diameter, the presence of possible CLNM in advance by preoperative ultrasound, the presence of CLT and LMR were independent risk factors for preoperative CLNM in patients with solitary PTCI, whereas age, isthmus tumor location, the presence of CLT, gross tumor diameter, the presence of intraoperative ETE, and the presence of Delphian lymph node metastasis (DLNM) in the frozen section (FSE) were independent risk factors for the intraoperative development of CLNM. On this basis, we established preoperative and intraoperative CLNM prediction nomograms (Fig. 2a/b).

**Table 3:** Univariate analysis and multivariate analysis of preoperative independent predictors for CLNM in solitary PTCI patients

Variables	Preoperative model				
	Univariate analysis		*P* value	Multivariate analysis	*P* value
	With CLNM (N = 121)	Without CLNM (N = 120)		Adjusted OR (95% CI)	
Age (years)			< 0.001		
≤ 40	55 (45.5%)	23 (19.2%)		1	
> 40	66 (54.5%)	97 (80.8%)		0.235 (0.108–0.508)	< 0.001
Sex			0.001		
Female	87 (71.9%)	107 (89.2%)		1	
Male	34 (28.1%)	13 (10.8%)		4.682 (1.918–11.427)	0.001
BMI			0.463		
Normal	73 (60.3%)	76 (63.3%)			
Overweight	37 (30.6%)	38 (31.7%)			
Obesity	11 (9.1%)	6 (5.0%)			
CLT			0.001		
Absence	99 (81.8%)	74 (61.7%)		1	
Presence	22 (18.2%)	46 (38.3%)		0.266 (0.123–0.576)	0.001
Maximum tumor size			< 0.001		
≤ 0.4	2 (1.7%)	13 (10.8%)		1	
(0.4–0.7]	30 (24.8%)	66 (55.0%)		1.906 (0.341–10.651)	0.462
(0.7–1]	41 (33.9%)	32 (26.7%)		7.378 (1.289–42.218)	0.025
> 1	48 (39.7%)	9 (7.5%)		32.707 (5.081–210.530)	< 0.001
Tumor volume			< 0.001		
M(IQR)	0.27 (0.11,0.58)	0.08 (0.04,0.16)			
Location			< 0.001		
Paraisthmic tumor	64 (52.9%)	96 (80.0%)		1	
Median isthmic tumor	57 (47.1%)	24 (20.0%)		2.538 (1.201–5.361)	0.015
Probable CLNM detected during US			< 0.001		
Absence	95 (78.5%)	116 (96.7%)		1	
Presence	26 (21.5%)	4 (3.3%)		4.828 (1.288–18.094)	0.02
ETE detected during US			0.011		
Absence	110 (90.9%)	118 (98.3%)			
Presence	11 (9.1%)	2 (1.7%)			
PLR			0.281		
Mean (SD)	136.77 (46.68)	142.96 (44.16)			
NLR			0.143		
Mean (SD)	1.76 (0.65)	1.91 (0.90)			
LMR			0.036		
Mean (SD)	4.96 (1.14)	5.34 (1.64)		1.312 (1.044–1.648)	0.02
SII			0.093		
Mean (SD)	431.95 (181.16)	477.64 (235.80)			
Dyslipidemia			0.954		
Absence	54 (44.6%)	54 (45.0%)			
Presence	67 (55.4%)	66 (55.0%)			
Creatinine			0.560		
M(IQR)	58.10 (52.95, 69.20)	58.60 (53.63, 63.98)			
Tg			0.090		
M(IQR)	16.70 (7.32, 36.50)	15.20 (5.32, 22.77)			
TSH			0.392		
M(IQR)	2.94 (1.89, 4.01)	2.71 (1.70, 3.93)			

*CLNM* central lymph node metastasis, *PTCI* papillary thyroid carcinoma of the isthmus, *BMI* body mass index, *CLT* chronic lymphocytic thyroiditis, *US* ultrasound, *ETE* extrathyroidal extension, *DLNM* Delphian lymph node metastasis, *PLR* platelet/lymphocyte ratio, *NLR* neutrophil/lymphocyte ratio, *LMR* lymphocyte/monocyte ratio, *SII* the systemic immune-inflammation index, *Tg* Thyroglobulins, *TSH* thyroid stimulating hormone

**Table 4:** Univariate analysis and multivariate analysis of intraoperative independent predictors for CLNM in solitary PTCI patients

Variables	Intraoperative model				
	Univariate analysis		*P* value	Multivariate analysis	*P* value
	With CLNM (N = 114)	Without CLNM (N = 127)		Adjusted OR (95% CI)	
Age (years)			< 0.001		
≤ 40	54 (47.4%)	24 (18.9%)		1	
> 40	60 (52.6%)	103 (81.1%)		0.189 (0.087–0.410)	< 0.001
Sex			0.001		
Female	82 (71.9%)	112 (88.2%)			
Male	32 (28.1%)	15 (11.8%)			
BMI			0.300		
Normal	67 (58.8%)	82 (64.6%)			
Overweight	36 (31.6%)	39 (30.7%)			
Obesity	11 (9.6%)	6 (4.7%)			
CLT			0.001		
Absence	93 (81.6%)	80 (63.0%)		1	
Presence	21 (18.4%)	47 (37.0%)		0.246 (0.106–0.572)	0.001
Gross maximum tumor size			< 0.001		
M(IQR)	0.70 (0.50, 1.00)	0.50 (0.40, 0.70)		13.022 (3.340–50.770)	< 0.001
Location			< 0.001		
Paraisthmic isthmic tumor	60 (52.6%)	100 (78.7%)		1	
Median isthmic tumor	54 (47.4%)	27 (21.3%)		2.514 (1.151–5.489)	0.021
ETE detected during surgery			< 0.001		
Absence	62 (54.4%)	105 (82.7%)		1	
Presence	52 (45.6%)	22 (17.3%)		4.529 (2.047–10.022)	< 0.001
DLNM			< 0.001		
Absence	86 (75.4%)	123 (96.9%)		1	
Presence	28 (24.6%)	4 (3.1%)		5.791 (1.803–18.599)	0.003
PLR			0.183		
Mean (SD)	135.82 (44.41)	143.47 (44.33)			
NLR			0.18		
Mean (SD)	1.77 (0.66)	1.90 (0.88)			
LMR			0.088		
Mean (SD)	4.99 (1.13)	5.29 (1.63)		1.268 (0.008–1.600)	0.045
SII			0.111		
Mean (SD)	431.83 (183.07)	475.23 (231.97)			
Dyslipidemia			0.813		
Absence	52 (45.6%)	56 (44.1%)			
Presence	62 (54.4%)	71 (55.9%)			
Creatinine			0.370		
M(IQR)	58.10 (53.02, 70.15)	58.5 (53.5, 63.9)			
Tg			0.096		
M(IQR)	16.93 (7.23, 37.00)	2.72 (1.70, 3.98)			
TSH			0.632		
M(IQR)	2.90 (1.85, 3.92)	15.32 (5.73, 22.06)			

*CLNM* central lymph node metastasis, *PTCI* papillary thyroid carcinoma of the isthmus, *BMI* body mass index, *CLT* chronic lymphocytic thyroiditis, *ETE* extrathyroidal extension, *DLNM* Delphian lymph node metastasis, *PLR* platelet/lymphocyte ratio, *NLR* neutrophil/lymphocyte ratio, *LMR* lymphocyte/monocyte ratio, *SII* the systemic immune-inflammation index, *Tg* Thyroglobulins, *TSH* Thyroid stimulating hormone

### Internal validation and external validation of the predicted nomograms

**Fig. 3:**
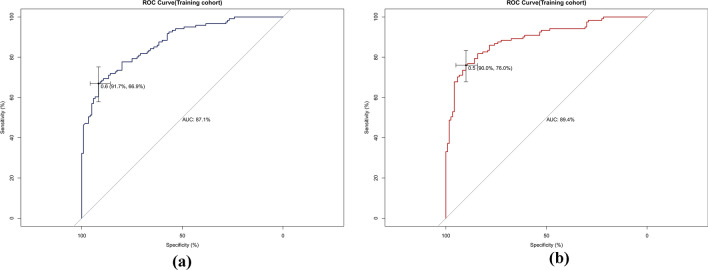
**a** The ROC curve in the training set for the preoperative model; **b** the ROC curve in the training set for the intraoperative model

**Fig. 4:**
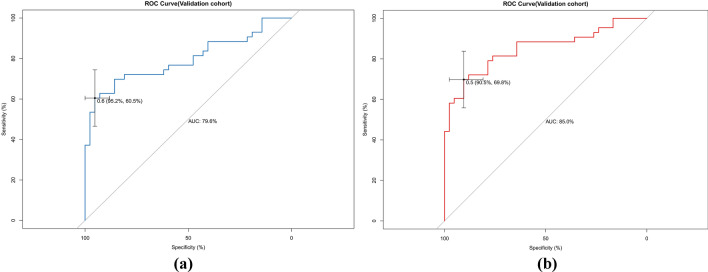
**a** The ROC curve in the validation set for the preoperative model; **b** the ROC curve in the validation set for the intraoperative model

**Fig. 5:**
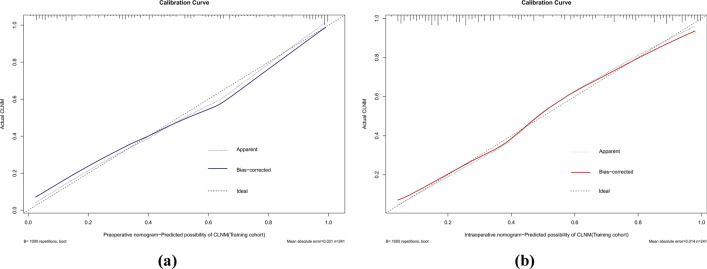
**a** The calibration curve in the training set for the preoperative model; **b** the calibration curve in the training set for the intraoperative model

The AUCs for the training set were 0.871 (95% CI 0.828–0.914) and 0.894 (95% CI 0.853–0.934) for the preoperative and intraoperative models (Fig. 3a**/**b), respectively, while the corresponding AUCs for the validation set were 0.796 (95% CI 0.698–0.895) and 0.851 (95% CI 0.765–0.938) (Fig. 4a/b). The calibration curve (Fig. 5a/b, 6a/b) and DCA curve (Fig. 7a/b, 8a/b) of this study both performed well, indicating that the model has good predictive performance and clinical applicability. In addition, we compared each independent risk factor with the established model in the ROC curve and DCA curve, and it was observed that nomograms had better predictive performance than individual factors. (Fig. 9a/b, 10a/b).

**Fig. 6:**
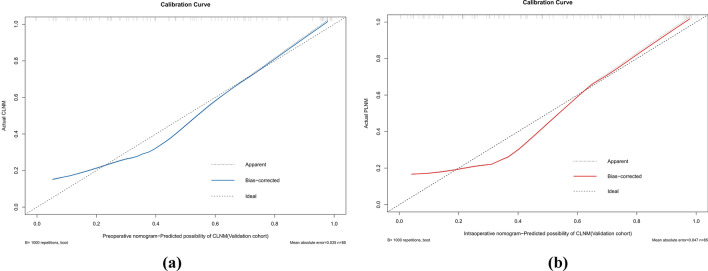
**a** The calibration curve in the validation set for the preoperative model; **b** the calibration curve in the validation set for the intraoperative model

**Fig. 7:**
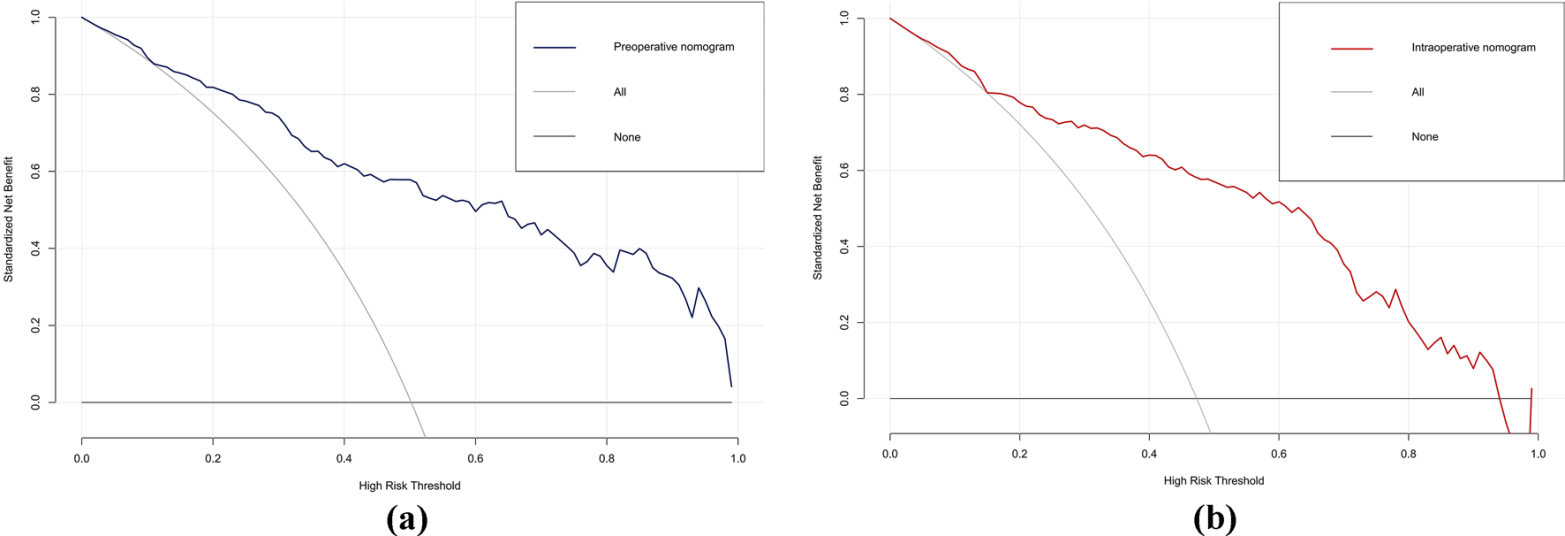
**a** The DCA curve in the training set for the preoperative model; **b** the DCA curve in the training set for the intraoperative model

**Fig. 8:**
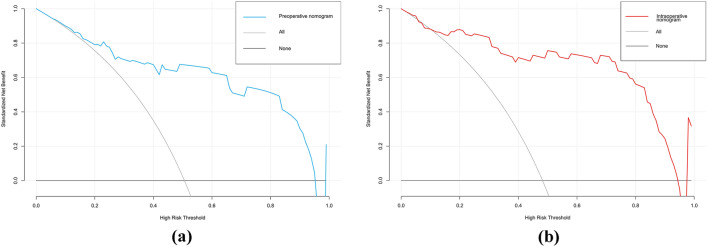
**a** The DCA curve in the validation set for the preoperative model; **b** the DCA curve in the validation set for the intraoperative model

**Fig. 9:**
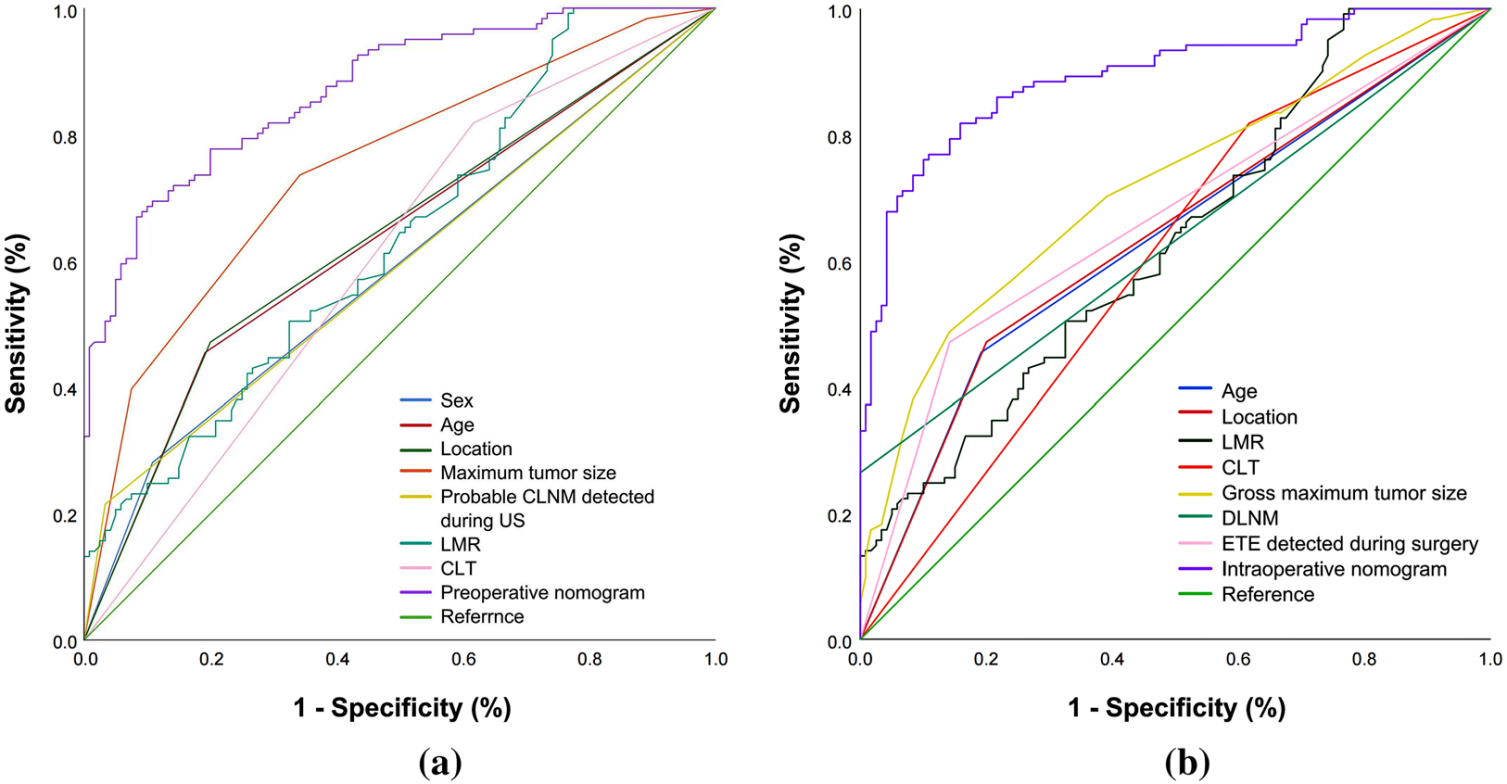
Each independent risk factor with the established model in the one ROC curve: **a** the preoperative model; **b** the intraoperative model

**Fig. 10:**
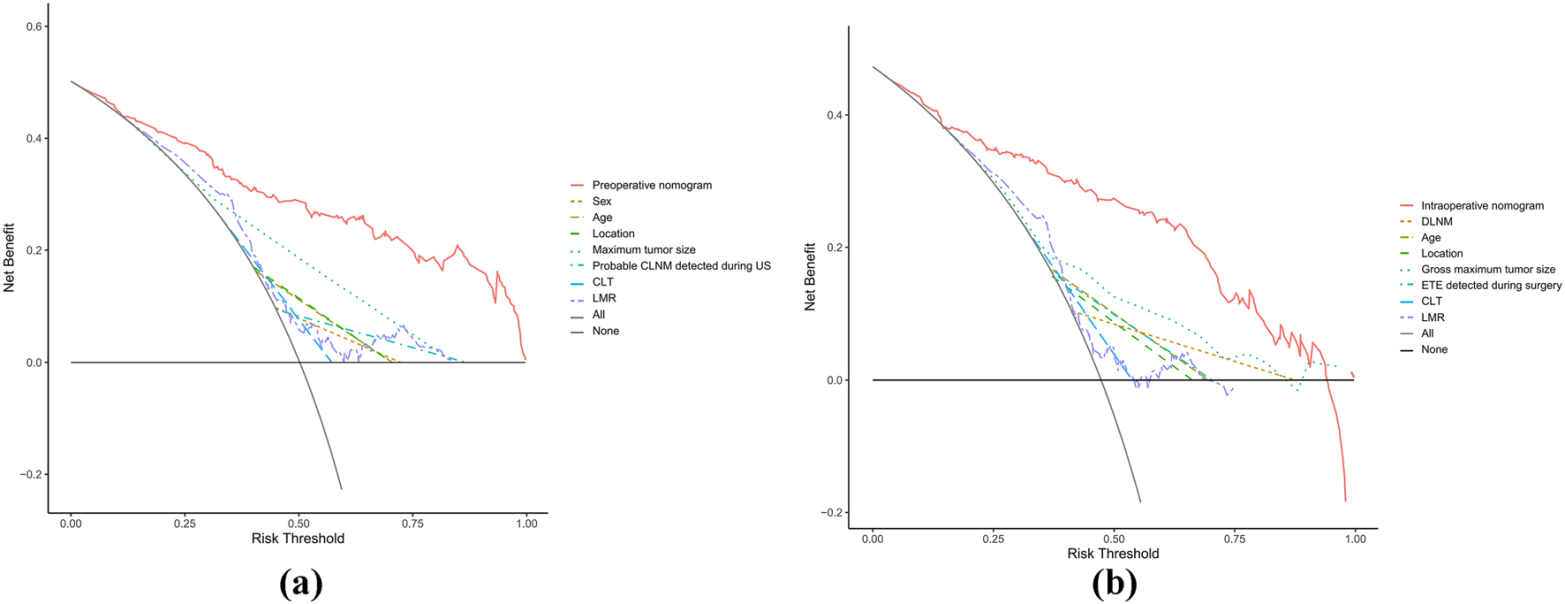
Each independent risk factor with the established model in the one DCA curve: **a** the preoperative model; **b** the intraoperative model

### CLNM risk stratification based on predictive nomograms

By summing the risk scores corresponding to the different variables in the preoperative and intraoperative nomograms to calculate the total score, we can predict the probability of CLNM occurrence. As shown in Table 5, we selected three cutoff values based on the risk scores, thus dividing them into four subgroups, i.e., low-risk group, medium-risk group, high-risk group, and extremely high-risk group. In the training set, the actual incidence of CLNM in the preoperative nomograms for each group was 93.6%, 63.6%, 34.4%, and 9.6% (*P* < 0.001), and the actual incidence of CLNM in the intraoperative nomograms corresponded to 88.6%, 65.7%, 25.0%, and 12.1% (*P* < 0.001). The incidence of total scores obtained by preoperative nomograms in the validation group was 95.8%, 53.8%, 30.75%, and 22.7% (*P* < 0.001) for each group according to this division criterion, and 100.0%, 62.9%, 13.6%, and 21.0% (*P* < 0.001) for intraoperative nomograms.

**Table 5:** Metastasis risk stratification of patients with PTCI based on risk scores of preoperative and intraoperative model

			LR	MR	HR	EHR	Total	*P* value
Training set	Preoperative model	Scoring range	< 261	[261,285)	[285,311)	≥ 311		
		With CLNM	6 (9.7%)	21 (34.4%)	35 (63.6%)	59 (93.6%)	121	
		Without CLNM	56 (90.3%)	40 (65.6%)	20 (36.4%)	4 (6.3%)	120	
		Total	62	61	55	63		< 0.001
	Intraoperative model	Scoring range	< 228	[228,254)	[254,297)	≥ 297		
		With CLNM	8 (12.1%)	13 (25.0%)	46 (65.7%)	47 (88.7%)	114	
		Without CLNM	58 (77.9%)	39 (75.0%)	24 (34.3%)	6 (11.3%)	127	
		Total	66	52	70	53		< 0.001
Validation set	Preoperative model	With CLNM	5 (22.7%)	8 (30.8%)	7 (53.8%)	23 (95.8%)	43	
		Without CLNM	17 (77.3%)	18 (69.2%)	6 (46.2%)	1 (4.2%)	42	
		Total	22	26	13	24		< 0.001
	Intraoperative model	With CLNM	4 (21.1%)	3 (13.6%)	17 (63.0%)	17 (100.0%)	41	
		Without CLNM	15 (78.9%)	19 (86.4%)	10 (37.0%)	0 (0.0%)	44	
		Total	19	22	27	17		< 0.001

*PTCI* papillary thyroid carcinoma of the isthmus, *LR* low-risk, *MR* moderated-risk, *HR* high-risk, *EHR* extreme high-risk, *CLNM* central lymph node metastasis

## Discussion

The isthmus of the thyroid gland is often located anterior to the cartilaginous ring of the 2nd to 4th trachea, where the gland thins to a thickness of only about 2 mm. The incidence of PTCI is approximately 2.5–9.2% (Lyu et al. 2021) and 6.78% in the present study. This discrepancy may be due to the inclusion of multifocal PTCI in previous studies and the controversy over the definition of the exact location of PTCI in the academic community (Zhu et al. 2023).

Due to the specificity of the tumor location, PTCI is more aggressive compared to lobar PTC. It was found that the probability of multifocal appearance of PTCI is higher compared to PTC in other locations (Lee et al. 2010), which makes it more likely that ETE, LNM, and vascular invasion will occur, factors that often suggest a poor prognosis. For this reason, this study excluded multifocal PTCI and studied only solitary PTCI with relatively reduced invasiveness and more controversial surgical extent. It has been shown that when the tumor diameter is > 0.7 cm, PTCI is more likely to break through the capsule, and due to the thinness of the isthmus, it is easy to invade perithyroidal tissues such as muscle or trachea after breaking through the capsule, i.e., ETE occurs (Luo et al. 2020). In this study, ETE was found intraoperatively in 100 patients (30.7%), of which 45 (45%) occurred in the median isthmus. Bortz et al. (Bortz et al. 2021) suggested that both microscopic ETE and visual ETE increase the likelihood of LNM and distant metastases, and the ETE found intraoperatively in this study became an independent risk factor for the development of CLNM in patients with PTCI. However, some studies have also found that ETE is not a risk factor for the development of LNM in PTCI (Seok et al. 2020) and that microscopic ETE does not have an impact on the prognosis of patients, the exact mechanism of which is not clear (Park et al. 2020).

The presence of the above pathological features makes PTCI more prone to CLNM and shows a unique metastatic pattern (Lyu et al. 2021), which may be due to the abundant lymphatic vessels between the thyroid peritoneal tissues making PTCI more prone to LNM compared to lobar PTC, with a CLNM rate of 33.6% for lobar PTC in this study, which is lower than the 50.3% for PTCI. In addition, the specific location of the isthmus tumor was also concluded to be an independent risk factor for the occurrence of CLNM in this study, and median PTCI was more prone to CLNM preoperatively and intraoperatively compared to paracentral PTCI, which is consistent with the findings of Feng et al. (2020). Notably, Gui et al. (2020) found no significant correlation between the specific location of the isthmus tumor and its CLNM, which may be due to differences in the definition of the extent of the isthmus in different studies. Although the presence of CLNM should be suspected when ultrasound suggests abundant lymph node blood flow with cystic changes, eccentric solid hyperechoic masses and gravel-like calcifications in the lymph nodes, or the disappearance of lymphatic portals (Leboulleux et al. 2007), the diagnostic sensitivity of ultrasound for CLNM is low due to the possible interference of intratracheal gas with ultrasound examination, and only 22.5% of patients with CLNM in this study PTCI patients with preoperative ultrasound suggestive of CLNM, so it should be evaluated together with other preoperative indicators for the risk of CLNM. Generally, the sequence of LNM in the majority of PTCI patients is from central to lateral cervical lymph nodes, with jumping metastases occurring less frequently. Zhou et al. (2020) suggested that even in patients with the same sex, BRAF V600E mutation status, tumor size, ETE status and pathological TNM stage, the pretracheal and bilateral paratracheal lymph node metastasis rates were higher in PTCI than in lobar PTC (pretracheal LNM: 35.6 vs. 13.8%; bilateral paratracheal LNM: 24.1 vs. 6.9%). Zhao et al. (2022) suggested that DLN may play a “prodromal” role in the metastasis of PTCI lymph nodes, and that DLNM increases the likelihood of LLNM and bilateral CLNM. In this study, the incidence of DLNM was 14.4%, and FSE returned DLNM as an independent risk factor for CLNM. Forty-two patients (89.3%) with DLNM had pretracheal and paratracheal lymph node metastases, suggesting that the scope of surgery can be decided intraoperatively based on the FSE results of DLN. In addition, the metastasis rate of the lymph node posterior to the right recurrent laryngeal nerve (LN-prRLN) in this study was 5.5%, which is similar to the findings of Du et al. (2021) (6.4%), which concluded that tumor diameter ≥ 0.5 cm, multifocality, ETE, and the presence of other LNM are independent risk factors for the development of metastasis in the LN-prRLN in patients with PTCI, especially when the lymph node anterior to the right recurrent laryngeal nerve (LN-arRLN) metastasizes. Therefore, if intraoperative FSE results of the LN-arRLN do not show metastasis and there are no other adverse pathologic features, further clearance of the LN-prRLN may not be considered to minimize the stretching irritation of the laryngeal nerve.

Nevertheless, the extent of surgical resection for PTCI is not detailed in the relevant guidelines (Haugen et al. 2016; Horiguchi et al. 2021). One study reported the probability of temporary or permanent hypoparathyroidism and temporary laryngeal recurrent nerve injury after TT + BCLND to be 28.2% and 4.2%, respectively (Zhao et al. 2017), and the probability of temporary and permanent hypoparathyroidism after TT + UCLND in PTCI patients in our study was 30.4% and 2.5%, respectively. It has been suggested that extended isthmus resection + DLN and pretracheal lymph node dissection is also a better option for patients with PTCI, as it eliminates the need for dissection of the posterior thyroid capsule and exploration of the tracheoesophageal sulcus, thus avoiding postoperative complications such as hypocalcemia and hoarseness due to damage to the parathyroid glands and the laryngeal recurrent nerve. Seo et al. (2021) also concluded that approximately 90% of patients with PTCI without occult carcinoma may not require TT and concluded that isthmus resection is feasible for patients with small solitary cN0 stage PTCI without significant ETE, but patients should be informed of their potential risk of recurrence. Gui et al. (2020), by comparing three surgical procedures (TT, affected lobe thyroidectomy and isthmus, and isthmus resection), found no statistically significant difference in recurrence-free survival between the three groups and a significantly lower probability of adverse pathologic features such as LNM in patients with ≤ 1 cm diameter compared to those with solitary PTCI > 1 cm in diameter. Nevertheless, advances in surgeon techniques have led to a further reduction in the incidence of postoperative complications, and many studies have concluded that PTCI is highly invasive and TT + BCLND should be recommended (Li et al. 2017). The current academic approach to the surgical treatment of PTC is increasingly conservative, with the goal of minimizing the possible damage caused by surgery while ensuring the patient's prognosis. The long-term risk–benefit ratio of the procedure versus the conventional TT + BCLND procedure also needs to be further discussed.

Due to the relatively low incidence, previous studies on solitary PTCI have mostly focused on the analysis of its clinicopathological factors, and clinical prediction models for CLNM have been less developed. Feng et al. (2020) concluded that gender, BRAF V600E mutation, CLT, tumor size, specific tumor location, intraoperative detected ETE and BMI were independent risk factors for ipsilateral or contralateral CLNM in patients with PTCI, based on which a nomogram was constructed to assess the risk of developing CLNM in patients with PTCI. Although the model showed good discrimination and calibration, it was only internally validated due to the small number of cases included, and only ultrasound and pathological information of the patients were included in the study, without including other preoperative test results. In this study, we included routine preoperative serological test results to obtain more accurate and comprehensive risk factors associated with CLNM in patients with solitary PTCI. The relationship between tumor and inflammation is considered to be an important mechanism for tumor development, and a study by Huang et al. (2022a, b) concluded that blood inflammatory factors such as LMR, NLR, and PLR were not only significantly associated with recurrence in PTC patients, but also with clinicopathological features such as tumor size, LNM, and LNM rate. Our study also concluded that lower LMR was significantly associated with the development of CLNM in patients with PTCI, but did not conclude that creatinine value, hyperlipidemia, thyroglobulin, and thyroid stimulating hormone were associated with CLNM. In contrast, the clinical prediction model developed by Yang et al. (2020) regarding CLNM in patients with PTC incorporated creatinine values and achieved better predictive results, but the explanation of the associated mechanisms is not clear at this time.

The relationship between CLT and LNM has been controversial and no consensus has been reached on the exact mechanism. Meta-analysis of the relationship by Osborne et al. (2022) found that 11 of 22 studies did not find a statistical difference between the CLT and LNM, 8 studies observed a reduced probability of CLNM in patients with CLT, and only 1 study showed that CLT promotes the development of CLNM. The present study concluded from the analysis that CLT is a protective factor for CLNM in patients with solitary PTCI, which is consistent with the findings of Feng et al. (2020). In addition to this, age at diagnosis is a recognized prognostic factor for thyroid carcinoma survival, and in this study, we calculated the Jorden index for each patient by ROC curve and obtained the optimal age cutoff value of 40 years, and it is noteworthy that through analysis, we concluded that age at diagnosis > 40 years is a protective factor for the occurrence of CLNM in patients with solitary PTCI. Shukla et al. (2021) analyzed the SEER (surveillance, epidemiology, and end results) database and concluded that younger PTC patients are more likely to develop LNM and result in an increased risk of recurrence than older patients. The eighth edition of the AJCC DTC staging system increased the age cutoff from 45 to 55 years, but there is still controversy in the academic community regarding the determination of the age cutoff for assessing thyroid carcinoma patients' disease; some scholars believe that the cutoff value of 55 years improves the ability of the AJCC system to discriminate between disease-specific survival rates, but others say that the new criteria underestimate the staging of some younger patients (Kazaure et al. 2018).

Through internal validation and external validation, the preoperative and intraoperative nomograms in this study showed good performance in terms of discriminatory ability, calibration, and clinically applicable performance. In addition, we stratified patients for the risk of CLNM by the scores in the nomograms, and we recommend TT + BCLND for patients with preoperative or intraoperative ratings of extremely high-risk, high-risk, and median PTCI. For patients with low to moderate risk ratings and paracentral PTCI, we recommend first affected thyroid + isthmus resection + contralateral partial thyroidectomy + UCLND with FSE. If the results return the presence of LNM, the procedure should be further expanded. In the training set, there were 118 extremely high-risk and high-risk patients, and 19 patients were preoperatively low to moderate risk but intraoperatively high or extremely high-risk. The use of nomograms in the clinical setting can help surgeons to more accurately understand the disease progression of their patients to facilitate the development of individualized surgical plans for the solitary PTCI patients, thus avoiding overtreatment or even delayed treatment. Although the nomograms in this study has good predictive performance and clinical applicability, the study has some limitations. First, our study is retrospective and there is an inherent selection bias, such that the ultrasonographic diagnosis of thyroid nodules will inevitably be influenced by subjectivity. Second, the number of cases included in this study is still small, and future multicenter prospective studies and inclusion of more cases could be conducted to obtain more accurate and practical prediction models. Although this study divided the patient population according to the chronological order for validation, external validation from multiple centers is more convincing for model performance assessment and could be used in future studies. Finally, because this study included patient information for more than 5 years, during which time the diagnostic techniques for thyroid carcinoma have been improving, and in recent years, our institution has routinely performed preoperative BRAF V600E mutation detection by PCR on puncture specimens of suspicious nodes, which may differ from the results obtained by immunohistochemistry on postoperative pathology specimens in the past, we did not include molecular science such as BRAF V600E. However, the molecular field still has great value in guiding the diagnosis and treatment of thyroid carcinoma, and its inclusion is recommended for future studies.

## Conclusion

In summary, our study concluded that patients with solitary PTCI are more likely to develop adverse pathological features such as CLNM compared to solitary lobar PTC. The ROC curves, calibration curves, and DCA curves from internal and external validation showed that the resulting nomograms had excellent discrimination, calibration ability, and clinical applicability. In addition, we propose a risk stratification scheme for CLNM in patients with solitary PTCI to help surgeons develop individualized surgical plans for their patients.

## Data Availability

The datasets used and/or analyzed during the current study are available from the corresponding author on reasonable request.
